# The Type of Confinement (Outdoor Soil-Bedded vs. Compost Barn) Affects the Welfare of Autumn-Calving Dairy Cows Kept in Mixed-Feeding Systems

**DOI:** 10.1155/vmi/3527752

**Published:** 2025-07-03

**Authors:** M. V. Pons, M. L. Adrien, D. A. Mattiauda, M. N. Méndez, A. Meikle, P. Chilibroste, J. P. Damián

**Affiliations:** ^1^Departamento de Ciencias Veterinarias y Agrarias, Facultad de Veterinaria, Cenur Litoral Norte, Universidad de la República, Paysandú, Uruguay; ^2^Departamento de Producción Animal y Pasturas, Facultad de Agronomía, Universidad de la República, Paysandú, Uruguay; ^3^Laboratorio de Endocrinología y Metabolismo Animal, Departamento de Clínicas y Hospital Veterinario, Facultad de Veterinaria, Universidad de la República, Montevideo, Uruguay; ^4^Departamento de Biociencias Veterinarias, Facultad de Veterinaria, Universidad de la República, Montevideo, Uruguay; ^5^Núcleo de Bienestar Animal, Facultad de Veterinaria, Universidad de la República, Montevideo, Uruguay

## Abstract

In mixed systems (pasture plus TMR), it is essential to provide the cows with good housing conditions at times of supplementation during confinement. However, given that there are different types of confinement for high-producing cows in pastoral-based systems, it is necessary to determine how such confinement conditions can affect their welfare. The aim of this study was to determine if the type of confinement (compost barn (CB) versus outdoor soil-bedded (OD)) used in mixed systems affects the welfare of dairy cows through behavioral and biochemical indicators. Holstein cows (*n* = 32) calving in autumn were assigned to two treatments (CB or OD) at calving. During confinement (half a day) in CB, the cows were kept indoors with a compost bed (13.5 m^2^/cow), including ventilation, while OD cows were kept in the open space with a dirt floor and shaded area (4.8 m^2^/cow). Confinement and grazing behavior (eating, ruminating, drinking, lying, standing, and walking) and the number of agonistic interactions in confinement were recorded every 10 min, on 3 days within a week for 5 months. Blood was collected to determine the concentration of creatine kinase, total proteins, and albumin during lactation. During confinement, cows in CB were found to be more frequently eating (*p*=0.07), drinking (*p* < 0.0001), and lying down (*p*=0.003) than those in OD, while OD cows were more time ruminating (*p*=0.0005), standing (*p*=0.02), and walking (*p* < 0.0001). During pasture access, cows in CB were more time eating (*p*=0.01) and standing (*p*=0.0003), while they were less lying (*p*=0.01) than cows in the OD and no differences were found in ruminating, drinking, and walking behaviors. CB cows tended to present more agonist interactions than in OD cows (*p*=0.09). Cows in CB had higher total proteins (*p*=0.02) and globulins (*p*=0.006) than cows in OD. In conclusion, the type of confinement differentially affected the different behavioral indicators (lying, standing, walking, rumination, and agonist interactions), as well as blood concentration of total protein and globulins. Although CB did not have all the behavioral indicators of welfare in its favor for autumn-calving cows (ruminating and agonist interactions), blood indicators (total protein and globulins) highlight advantages of CB in relation to OD.

## 1. Introduction

Milk production systems that attempt to maximize pasture consumption have a high stocking rate with the use of supplementation; this is achieved by planning the calving season and applying a different feeding management [1, 2]. Autumn calving is used to prevent heat stress in early and midlactation when it has the greatest impact on milk production [3]. There are times when cows are unable to access pastures, due to weather conditions. For example, low temperatures in winter decrease the growth rate of pastures, and abundant rainfall makes access to grazing difficult, as well as generates the formation of large amounts of mud in heavily used by cows [4]. When these conditions are met cows are generally confined in the outdoor soil-bedded (OD) paddocks. In winter when there is heavy rainfall, these confinements are often muddy and waterlogged, and in some cases, the cows do not have a dry surface to lying [5, 6].

An alternative to mitigate the effects of unfavorable weather conditions can be partial housing in compost barn (CB) systems. This system consists of a compost bed of vegetable material (sawdust, wood chips, and rice husks) where compost is generated together with cow feces and urine [7]. CB systems have some advantages in favor of animal welfare; one of them is that they provide a more comfortable surface for cows compared with OD and free stall systems [7]. The housing systems most commonly seen in the region are the OD, also called dry lot or open lot, cows are provided with a small area with a roof to protect the animals from wind and rain in winter and high temperatures in summer [8]. In addition, they also have an area where troughs are located, which in some cases is also roofed, and with concrete floor or ballast to avoid mud accumulation in that area. In these conditions, cows may be more exposed to rainfall, with mud forming in the resting areas, mainly during fall and winter [5, 9].

When cows gain access to grazing, they can express the natural behaviors of the species, where 95% of their time is spent grazing, ruminating, and lying [10]. Of these behaviors, grazing is the most performed, followed by rumination and lying. Therefore, when deprivation of any of these natural behaviors occurs, the welfare of the dairy cow is compromised [11]. In terms of priority behaviors, if cows are denied the opportunity to lie down and feed and then given the opportunity to perform both behaviors, they prefer to spend more time lying down rather than feeding [11]. In mixed systems, it has been found that when cows are in confinement with mud on the ground, they avoid lying and then lie down in the pasture [5, 9, 12]. Cows tend to maintain a constant amount of resting time, which only change when environmental conditions for lying are not comfortable enough (excessive stocking density, wet bedding, and heat stress) as a short-term compensatory mechanism until conditions are suitable [11].

Aggressive behavior is also used as a welfare indicator, measured principally by the number of agonist interactions. In grazing, agonistic interactions are less than in confinement due to the greater availability of space available to the cows in the pasture [13]. Greater agonistic activity is observed when fresh feed is offered, and competition for feed increases as trough space per cow is reduced [14]. Therefore, to evaluate the behavior of cows in mixed systems, it is necessary to carry out a comprehensive evaluation, recording both the behaviors performed in the pen and on the pasture.

Animal welfare can be assessed using biochemical indicators, such as blood proteins. Among the proteins most frequently used as indicators of health and welfare are total proteins, albumin, globulins, creatine kinase, and acute phase proteins [15–17]. Acute phase proteins are blood proteins that are used to assess the nonspecific systemic response of the innate immune system to infection, inflammation, or trauma [18]. Different stress situations that negatively affect the welfare of the dairy cow such as abrupt change in feeding and housing [19], abrupt change of housing (tie-stall vs. free-stall housing) [20], stress of calving in dairy cows [21, 22], have generated changes in the concentrations of blood proteins. Therefore, blood proteins are important biochemical indicators of animal welfare.

Our group reported on the animal welfare advantages of CB vs. OD in mixed systems during the summer months. The CB cows were better able to cope with heat stress, as evidenced mainly by lower subcutaneous temperatures and higher frequency of lying during confinement, compared with the OD cows [16]. The results obtained previously show how spring-calving cows cope with adverse summer conditions, but it is necessary to know how autumn-calving cows adapt to autumn and winter conditions in CB and OD.

The hypothesis of this work was that in mixed systems (pasture plus total mixed ration (TMR)) dairy cows kept in CB present better welfare than those kept in OD during autumn and winter due to the better housing conditions in CB. Therefore, the objective of this study was to evaluate whether confinement in CB and OD affects behavioral and biochemical indicators in autumn-calving cows.

## 2. Materials and Methods

The experiment was carried out in the dairy farm of the Estación Experimental Dr. Mario A. Cassinoni (EEMAC), Facultad de Agronomía, Universidad de la República. The experimental protocol was approved by the Comisión de Ética en el Uso de Animales de la Facultad de Agronomía (ID 682-Exp020300-000602-18).

Thirty-two Holstein dairy cows (24 multiparous and 8 primiparous) calved in the autumn (03/17/2019 ± 9.6 days) were include into the experiment. Cows were blocked by lactation number, expected calving date, live weight, and body condition before calving and were randomly assigned to one of two groups immediately after calving. Average cow weight at calving was 632 ± 92 kg (means ± standard deviation) and body condition at calving was 2.90 ± 0.40 (means ± standard deviation, scale 1-5, [23]).

**Figure 1:**
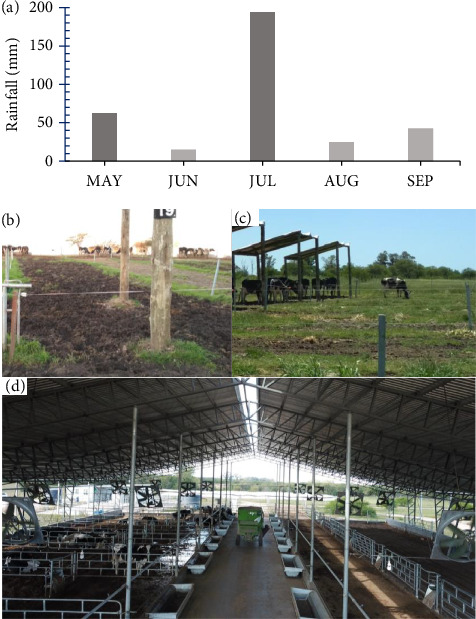
Cumulative rainfall (mm) during the 15 days prior to the behavioral evaluation (a) (MAY: May; JUN: June; JUL: July; AUG: August; and SEP: September). The dark gray bars indicate the presence of mud on the entire surface of the pens in the OD group during the behavioral evaluation (b), while the light bars indicate that the surface of the OD pen was free of mud (c). Confinement of cows from compost barn groups (d).

Cows had access to grazing for half a day and then were confined with supplementation (mixed systems) according to groups: (i) confinement in a roofed barn with compost bedding (CB) and (ii) an OD confinement with a shaded area (OD, Figure 1). The cows were in early lactation in autumn and winter and mid lactation in spring. The conditions provided during the confinement in both groups were described by Pons et al. [16]. In both groups, the animals were confined of 4 animals per pen. After calving, the cows entered their group and pen and remained in the group for 6 months. Cows were milked twice daily at 4:00 and 15:30 h.

### 2.1. Feeding

The cows grazed daily between 7:00 and 14:30. Pastures consisted of *Medicago sativa* and *Dactylis glomerata* (first year since implantation), grassland composed of *Festuca arundinacea*, *Trifolium repens*, and *Lotus corniculatus* (third year since implantation) or *Avena sativa*. The cows were grazed in a 7-day grazing plot. The grazing system had an annual stocking rate of 2.5 cows per hectare of grazing area and each group had its assigned area which was maintained throughout the experiment. The road from the pasture to the dairy facilities and enclosures was ballast and the distance between the confinement areas and the pastures varied between 1200 and 1500 m. Supplementation was administered in the form of TMR in feeders for each pen (CB: 0.5 m/cow and OD: 0.75 m/cow) 1 hour after entering the confinement and cows consumed all the supplements offered. Cows in both groups consumed daily the same TMR mixture composed of 56% concentrate and 44% forage.  Table 1 shows supplement intake, pasture availability, and allowance for each group in the months evaluated.

### 2.2. Behavior

The cows were painted with a visible number on the scapular area and the group on both sides to observe the individual behavior of each cow. Feeding behaviors (eating, ruminating, and drinking) and activity (lying, standing, and walking) were evaluated by visual observation in scanning every 10 min, on 3 days within a week and in each month [16]. All observers were previously trained and one observer was assigned to each group for simultaneous observations. Behaviors were assessed over 5 months (May–September) and from 43 days in lactation on average. Behavioral observations in confinement were made from 16:30 to 22:30 h (confinement: 15:30–4:00 h) and in grazing from 7:00 to 14:00 h (during the whole grazing period). During the month of May, no behavior was performed during access to the pasture. The percentage of observations of each behavior was calculated according to the number of times each cow performed that behavior in the total number of observations during the 6 h of evaluation [16, 24].

Agonistic behavior was observed continuously for 1 hour during confinement immediately after feeding of the animals during the same period as individual behavior. The behaviors recorded were bunting, pushing, and forceful, nonforceful, and head–head contacts as reported by Dickson et al. [25]. The number of interactions per cow was counted continuously for 1 h, in addition to recording the location where the interaction took place (trough, bedding, and drinking trough). To assess agonist interactions, observers were trained for one day to evaluate and classify the different agonist interactions, with pictures and definitions of each.

### 2.3. Blood Collection and Blood Protein Determination

Blood samples were collected from the coccygeal vein (without anticoagulant) after the morning milking. Samples were collected weekly until 120 days of lactation and biweekly from 120 days to the end of lactation. After each sample collection, the tubes were centrifuged at 3000 rpm for 10 min. The serum was placed in eppendorfs and identified with the date and cow number and stored at −20°C until further analysis. The samples that were closest to the behavioral evaluation dates were selected for analysis of creatine kinase, total protein, and albumin. Globulin concentration was estimated by the difference between total protein and albumin [24]. Blood proteins were determined by spectrophotometry (BA200, Biosystems S.A, Barcelona, España), at the Laboratorio de Endocrinología y Metabolismo Animal, Facultad de Veterinaria, Montevideo, Uruguay. Commercial kits from Biosystems S.A, Barcelona, España, were used. For all cases, the interassay CV of the commercial control sera was ≤ 10%.

### 2.4. Rainfall

Daily precipitation was obtained from weather station records within the experimental station. Figure 1(a) shows the accumulated precipitation in the 15 days prior to the behavioral evaluation. The months in which mud was present on the entire surface of the pens in the OD group associated with the accumulated precipitation were recorded (Figures 1(a), 1(b), and 1(c)).

### 2.5. Statistical Analysis

Data were analyzed by the procedure GLIMMIX of SAS OnDemand for Academics (v. 3.1.0, SAS Institute Inc., Cary, NC, USA). A repeated measurements analysis of variance was performed on the different variables. For the statistical analysis, the type of distribution of the variables was considered in the GLIMMIX procedure. The fixed effects for the behavior and blood proteins were group (CB vs. OD), month, and their interactions. For individual behavior and blood proteins, the animal (experimental unit) within each group and day was considered a random effect. For agonistic behavior, the pen (experimental unit) within each group and day was considered a random effect. An autoregressive covariance structure of order 1 was used. The results are expressed as the mean ± standard error of the mean (SEM). Differences were considered significant with an *p* values ≤ 0.05. A trend was considered when *p* values were between 0.05 and 0.10.

## 3. Results

**Figure 2:**
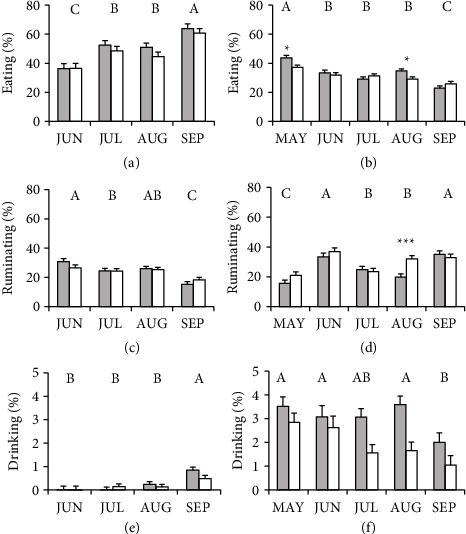
Frequency (mean % of observations ± SEM) of eating (a-b), ruminating (c-d), and drinking (e-f) behaviors during pasture access (left) and confinement (right) of Holstein dairy cows in compost barn (gray bars, *n* = 16) or in outdoor soil-bedded (white bars, *n* = 16) in May (MAY), June (JUN), July (JUL), August (AUG), and September (SEP). Different letters indicate significant differences between months (*p* < 0.05). Asterisks indicate significant differences between groups within the same month (interaction group x month, ^∗^*p* < 0.05; ^∗∗∗^*p* < 0.001).

During confinement, cows in CB tended to eating more frequently than those in OD (Table 2). There was a significant effect of month and group × month interaction for eating behavior (Table 2). Cows in CB presented higher frequency of eating behavior than cows in OD in the months of May and August (Figure 2(b)). The average eating behavior of both groups decreased across months, with the highest value observed in May and the lowest value in September (MAY: 40.4 ± 1.30%; JUN: 32.6 ± 1.41%; JUL: 30.2 ± 1.22%; AUG: 31.9 ± 1.22%; and SEP: 24.4 ± 1.30%; Figure 2(b)). During pasture access, cows in CB were grazing more than cows in OD (Table 2). Differences were found in this behavior along the months, but the interaction (group × month) was not significant (Table 2). From June to September, the average eating behavior of both groups was increasing, with the lowest value recorded in June and the highest value in September (JUN: 36.5 ± 3.03%; JUL: 50.6 ± 2.81%; AUG: 47.8 ± 2.80%; and SEP: 62.3 ± 2.90%; Figure 2(a)).

The frequency of rumination during confinement was higher in cows in OD than that in cows in CB (Table 2). In the month of August, cows in OD ruminated more than those in CB (Figure 2(d)). The rumination behavior varied throughout the months; rumination frequency (average of both groups) increased from May (18.2 ± 2.11%) to June (35.1 ± 2.24%), then decreased in July (24.1 ± 2.03%) and August (25.9 ± 2.03%), and increased again in September (33.9 ± 2.13%, Figure 2(d)). During access to the pasture, no effect of group or group × month interaction was found on rumination behavior, while differences were found in the average behavior of both groups over the months (Table 2, JUN: 28.7 ± 1.62%; JUL: 24.5 ± 1.39%; AUG: 25.7 ± 1.39%; and SEP: 16.8 ± 1.49%; Figure 2(c)).

Cows in the CB group drank water more frequently than those in the OD group during confinement, while there was no group × month interaction (Table 2). During the first 4 months, the frequency of this behavior was maintained (MAY: 3.2 ± 0.29%; JUN: 2.9 ± 0.34%; JUL: 2.3 ± 0.26%; and AUG: 2.6 ± 0.26%), but this decreased in the last month (SEP: 1.5 ± 0.29%; Figure 2(f)). In the pasture, no effect of group or group × month interaction was found on drinking behavior (Table 2). However, there was an effect of the month over the water drinking frequency and was highest in September (JUN: 0.00 ± 0.12%; JUL: 0.07 ± 0.09%; AUG: 0.18 ± 0.09%; and SEP: 0.66 ± 0.10%; Figure 2(e)).

**Figure 3:**
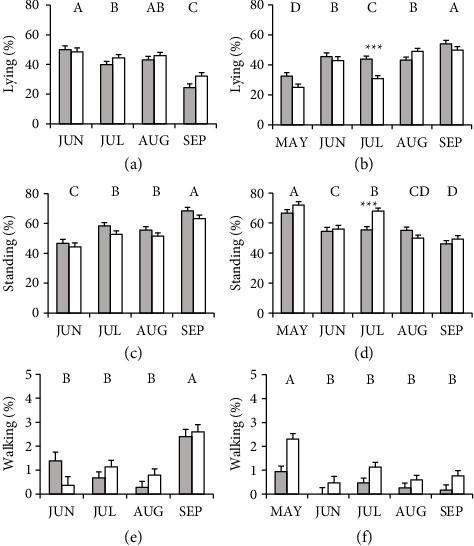
Frequency (mean % observations ± SEM) of lying (a-b), standing (c-d), and walking (e-f) behaviors during pasture access (left) and confinement (right) of Holstein dairy cows with partial confinement in compost barn (gray bars, *n* = 16) or outdoor soil-bedded (white bars, *n* = 16) in the months of May (MAY), June (JUN), July (JUL), August (AUG), and September (SEP). Different letters indicate significant differences between months (*p* < 0.05). Asterisks indicate significant differences between groups within the same month (interaction group x month, ^∗∗∗^*p* < 0.001).

Lying behavior during confinement was affected by group, month, and group × month interaction. A higher frequency of lying was found in cows in CB during confinement than in cows in OD (Table 2). Cows in CB were lying out more frequently than those in OD during the month of July (Figure 3(b)). The frequency of lying was different across months for cows in both groups (MAY: 28.7 ± 1.82%, JUN: 44.1 ± 2.00%; JUL: 37.2 ± 1.68%, AUG: 46.0 ± 1.68%; and SEP: 51.9 ± 1.82%; Figure 3(b)). In the pasture, cows in CB were lying less frequently than cows in OD (Table 2). There was no group × month interaction. The average frequency of lying in cows of both groups decreased from June to September (JUN: 49.2 ± 2.23%; JUL: 42.2 ± 1.9%; AUG: 44.6 ± 1.90%; and SEP: 28.3 ± 2.04%; Figure 3(a)).

Cows in OD were standing more frequently than those in CB during confinement (Table 5). In the month of July, a higher frequency of standing was found for cows in OD compared with cows in CB (Figure 3(d)). Cows in both groups (on average) were standing more frequently in May, but in June, the frequency decreased compared with May and increased in July and later decreased in August and September (MAY: 69.3 ± 1.80%; JUN: 55.2 ± 1.99%; JUL: 61.7 ± 1.66%; AUG: 52.5 ± 1.66%; and SEP: 47.6 ± 1.80%; Figure 3(d)). During pasture, cows in OD were less standing than cows in CB (Table 2). No group × month interaction was found for this behavior (Table 2). The frequency of standing on average of both groups increased from June to September (JUN: 45.4 ± 2.34%; JUL: 55.5 ± 2.05%; AUG: 53.5 ± 5.05%; and SEP: 65.7 ± 2.16%; Figure 3(c)).

Cows in OD walked more frequently during confinement than cows in CB (Table 2). No group × month interaction was found for walking (Table 2). A higher average frequency was observed in both groups in the month of May compared with the other months (MAY: 1.6 ± 0.17%; JUN: 0.2 ± 0.20%; JUL: 0.8 ± 0.14%; AUG: 0.4 ± 0.14%; and SEP: 0.5 ± 0.17%; Figure 3(f)). During access to the pasture, no differences were found between groups and no effect of the group × month interaction was found (Table 2). In September, the cows of both groups walked more frequently than in the other months (Table 6) (JUN: 0.9 ± 0.27%; JUL: 0.9 ± 0.20%; AUG: 0.5 ± 0.20%; and SEP: 2.5 ± 0.22%; Figure 3(e)).

**Figure 4:**
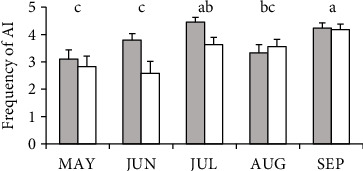
Frequency of agonist interactions (AIs) of Holstein dairy cows during confinement in compost barn (gray bars, *n* = 16) or outdoor soil-bedded (white bars, *n* = 16) in May (MAY), June (JUN), July (JUL), August (AUG), and September (SEP). Different letters indicate significant differences between months (*p* < 0.05).

In the other hand, during confinement, cows in CB tended to have more agonist interactions than cows in OD (CB: 3.8 ± 0.13 and OD: 3.4 ± 0.16; *p*=0.09). No effect of group × month interaction was found, but there was an effect of month (average of both groups, Figure 4).

**Figure 5:**
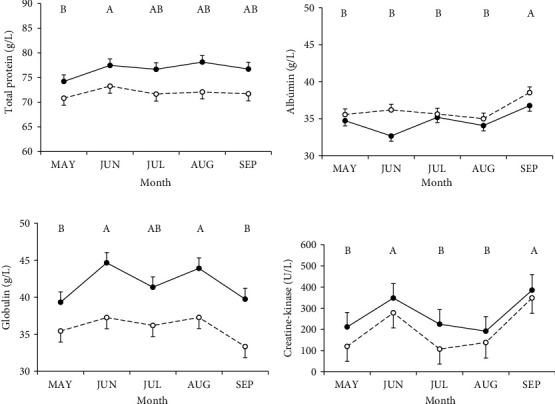
Concentration of total proteins (a), albumins (b), globulins (c), and creatine kinase (d) in Holstein dairy cows with partial confinement in compost barn (continuous line, *n* = 16) or outdoor soil-bedded (dashed line, *n* = 16) in May (MAY), June (JUN), July (JUL), August (AUG), and September (SEP). Different letters indicate significant differences between months (*p* < 0.05).

Serum concentrations of total proteins and globulins were higher in cows in CB than those in cows in OD, while albumin concentrations tended to be higher in cows in OD than in cows in CB (Table 3 and Figure 5). In serum creatine kinase concentrations, no significant differences were found between groups (Table 3 and Figure 5). No effect of group × month interaction was found, but there was an effect of month in total protein, albumin, globulin, and creatine kinase (average of both groups, Figure 5).

## 4. Discussion

In this study, it was observed that the different confinement conditions (CB vs. OD) affected the welfare of cows in mixed systems (grazing plus TMR), as evidenced by behavioral variables in confinement (all variables), in pasture (i.e., eating, lying, and standing) and biochemical (i.e., serum concentrations of total protein, albumin, and globulins) indicators. Based on the results of our study with fall calved cows, it was interesting to note that although the biochemical indicators were overall in favor of CB, the behavioral indicators of welfare were not always in favor of CB confinement. These results differ from those reported by our group under similar conditions, but with cows calving in spring [16], where cows in CB showed better welfare (including behavioral, biochemical, and physiological indicators). Therefore, this study, together with that reported by Pons et al. [16] highlights the importance of the calving season when evaluating the welfare of dairy cows under contrasting confinement conditions in mixed systems.

In this study, it was observed that cows in both groups varied in the frequency of certain behaviors and the changes varied between confinement and pasture. For example, CB cows were lying more than OD cows in confinement, while in pasture, this was reversed. This could indicate that CB cows chose to lie more on compost bedding, while OD cows preferred pasture. Related to this, preference studies show that cows prefer to lie on soft surfaces such as straw and pasture rather than sand or rubber [26, 27]. Rest is very important for welfare in dairy cows; the cows demonstrate a preference for lying on soft, dry surfaces. Cows prefer the resting surface provided by compost beds and pastures [10, 11, 28]. Conversely, when the resting site is wet and muddy (OD), it could affect resting conditions because the uncomfortable resting surface could prevent OD cows from expressing this behavior. In July (autumn month), when a large amount of mud was found in OD pens (Figure 1), OD cows decreased their lying behavior during confinement compared with CB cows. This result agrees with Chen et al. [5], who observed that lying was reduced by 75% when the floor was very muddy. On the other hand, the compost bedding provided a more comfortable resting surface for cows, which could also influence the fact that cows in CB were lying down more frequently compared with cows in OD [7]. However, during grazing, the cows in OD were lying more frequently than those cows in CB, perhaps to compensate for this behavior that they could not express during the OD confinement, or they preferred the softer surface to lie down on, such as the pasture. In addition, it is probable that by lying more in the pasture, the cows in OD were grazing less than the cows in CB. This compensatory strategy in relation to behavior has been reported by different authors. Fisher et al. [12] reported results similar to those found in this experiment, where the uncomfortable surface of the confinement decreased lying behavior, compensating with lying in the pasture. Tolkamp et al. [29] observed that as lying behavior is restricted, the likelihood of lying increases when more comfortable conditions are provided. Therefore, it is possible to speculate that different conditions during the stall led in cows OD to develop a different behavioral strategy than cows in CB. As a result, cows in CB lie down more frequently in confinement than cows in OD, given the comfortable conditions of the bedding. During pasture, this behavior is reversed, as cows in CB exhibit lying down behavior less frequently, while cows in OD take advantage of the pasture to lie down more, which was detrimental to grazing behavior.

The frequency of rumination during confinement in cows in CB was lower compared with OD cows. This difference occurs mainly in August, where a higher feeding frequency was also found in CB cows compared with OD cows. According to these results, the fact that CB cows ruminate for a lower proportion of time could be associated to that these animals eating more [30]. Cows in OD could have different strategies in performing their behavior during confinement than cows in CB, and these differences could also be associated with the differences found in grazing behavior. Eating and ruminating behavior is mainly influenced by feeding management, feed intake, physicochemical characteristics of the diet, and the health status of the animals [31]. There is a compensatory relationship between eating and ruminating behavior, where cows that spend more time eating spend less time ruminating [32]. It has also been reported that increasing stocking density can decrease rumination time [33]; however, CB cows were managed at the recommended stocking density for these CB systems. Because of this, it is suggested that OD cows in the pasture by lying down more and eating less than CB cows needed to consume the pasture faster. With less time to graze, cows in OD had less opportunity to select pasture, which may have affected rumination behavior between OD and CB cows during confinement. Furthermore, although cows are not the most selective ruminants, they can still select the pastures they consume when given the possibility, and this has also been related to better welfare conditions [34].

The higher frequency of drinking and lower frequency of walking in CB cows during confinement could be due to the distance to access water in their respective pens or be associated with the cows eating more. OD cows had to walk more to access water, and probably because of this, they drank less frequently than CB cows. The greater space available in OD pens compared to CB pens should also be considered since it has been reported that when animals have more space in the pens, they walk more [35]. In this sense, it seems that the different environmental conditions during confinement in mixed systems determine how cows behave and adapt to the conditions of these management systems.

In addition to individual behavior, this study evaluated the social behavior associated with agonistic interactions. Regarding these behaviors and under the conditions described in this study, cows in CB tended to display more agonistic interactions than those in OD, highlighting a negative aspect for cows in CB confinement. In this sense, this study highlights the need to consider the negative aspect of agonistic interactions in CB confinements, which may be associated with animal density. Possibly, the fact that CB cows present more agonistic interactions is associated to the smaller space per animal, mainly in troughs, since all the agonistic interactions recorded were in troughs. Feeder front space per cow was 0.5 m/cow in CB and 0.75 m/cow in OD. These differences in trough space may also affect eating and ruminating behavior. This increased competition for feed may impair cows' feeding and resting behaviors, as they may spend more time at the front of the trough competing for feed [36]. In free stall, DeVries et al. [37] found that cows with access to 0.5 m/cow of trough front had higher agonistic interactions and lower feeding activity than those with 1 m/cow.

Total protein and globulin concentrations were lower in the OD group than in CB. These lower protein concentrations in OD with respect to CB could evidence an alteration in protein metabolism due to a higher level of stress in OD cows [38]. The CB cows have higher globulin concentrations, which could have positive effects on their health, and could be less susceptible to diseases [21]. On the other hand, OD cows could be immunosuppressed due to greater exposure to adverse conditions such as wind, rain, and mud. Therefore, biochemical indicators measured in serum, such as the higher content of globulins and total proteins of the cows in CB showed a better state of welfare than those in OD.

It should be considered that weather conditions vary from 1 year to another so that more adverse conditions could aggravate the negative effects on the OD group, which is the most exposed to adverse weather conditions. The importance of this work is that it objectively evidences that the behavior of the cows is modified according to the characteristics of the environment during confinement. This can be considered when designing facilities and planning where cows will be when they cannot access pasture. Future studies should evaluate other welfare indicators, as well as economically evaluate confinement alternatives to complement the results obtained in this study.

## 5. Conclusion

When evaluating welfare indicator behaviors (lying, standing, walking, rumination, and agonist interactions), these were affected by confinement conditions. CB cows rested more and ruminated less frequently than OD cows during confinement. With respect to biochemical indicators, CB cows showed a better state of welfare (greater concentrations of total proteins and globulins) than OD cows. Although CB did not have all the behavioral indicators of welfare in its favor for autumn-calving cows, blood indicators (biochemical measures) highlight advantages of CB in relation to OD.

## Figures and Tables

**Table 1:** Supplement intake, pasture type, availability, and pasture allowance of Holstein dairy cows (evaluated from May to September) with access to grazing and partial confinement in compost barn (CB) or outdoor soil-bedded (OD).

Month	Supplement consumption (kg DM/cow/day)	Pasture	CB		OD	
			Availability (kg DM/ha)	Allowance (kg DM/cow/day)	Availability (kg DM/ha)	Allowance (kg DM/cow/day)
MAY	14	*Festuca arundinacea*	1550	18	2200	18
JUN	14	*Avena sativa*	1900	27	2000	29
JUL	14	*F. arundinacea*	2500	18	2700	19
AUG	16	*Medicago sativa + Dactylis glomerata*	2000	14	2000	14
SEP	14	*A. sativa*	2500	18	2500	18

**Table 2:** Frequency (mean % observations ± SEM) of eating, ruminating, drinking, lying, standing, and walking behaviors during confinement and during access to pasture of Holstein dairy cows in a mixed system with partial confinement in compost barn (CB, *n* = 16) or outdoor soil-bedded (OD, *n* = 16) in May, June, July, August, and September.

	Group		SEM	*p* value		
	CB	OD		G	M	G × M
*Confinement*						
Eating (%)	32.8	31.1	1.10	0.07	< 0.0001	< 0.0001
Ruminating (%)	25.7	29.2	1.95	0.0005	< 0.0001	< 0.0001
Drinking (%)	3.1	1.9	0.21	< 0.0001	0.0004	NS
Lying (%)	43.8	39.4	1.47	0.003	< 0.0001	< 0.0001
Standing (%)	55.5	59.0	1.45	0.02	< 0.0001	< 0.0001
Walking (%)	0.4	1.1	0.12	< 0.0001	< 0.0001	NS

*Pasture access*						
Eating (%)	51.0	47.6	2.75	0.01	< 0.0001	NS
Ruminating (%)	24.2	23.7	1.32	NS	< 0.0001	0.10
Drinking (%)	0.3	0.2	0.08	NS	< 0.0001	NS
Lying (%)	39.4	42.7	1.80	0.01	< 0.0001	NS
Standing (%)	57.2	52.9	1.98	0.0003	< 0.0001	NS
Walking (%)	1.2	1.2	0.17	NS	< 0.0001	0.05

*Note:* G: group. M: month. G × M: interaction between group and month.

Abbreviation: NS, not significant.

**Table 3:** Serum blood protein concentrations (total protein, albumin, globulin, and creatine kinase) of Holstein dairy cows in a mixed system with partial confinement in compost barn (CB, *n* = 16) or outdoor soil-bedded (OD, *n* = 16) from May to September, 2019.

Blood concentration	Group		SEM	*p* value		
	CB	OD		G	M	G × M
Total protein (g/L)	76.6	71.9	1.10	0.02	0.003	NS
Albumin (g/L)	34.7	36.2	0.49	0.09	< 0.0001	0.06
Globulin (g/L)	41.8	35.9	1.04	0.006	< 0.0001	NS
Creatine kinase (U/L)	272.4	198.4	39.9	NS	0.003	NS

*Note:* G: group. M: month. G × M: interaction between group and month.

Abbreviation: NS, not significant.

## Data Availability

The data that support the findings of this study are available from the corresponding author upon reasonable request.
